# Autism spectrum disorder and Coffin–Siris syndrome—Case report

**DOI:** 10.3389/fpsyt.2023.1199710

**Published:** 2023-08-24

**Authors:** Luka Milutinovic, Roberto Grujicic, Vanja Mandic Maravic, Ivana Joksic, Natasa Ljubomirovic, Milica Pejovic Milovancevic

**Affiliations:** ^1^Clinical Department for Children and Adolescents, Institute of Mental Health, Belgrade, Serbia; ^2^Day Hospital for Psychotic Disorders, Institute of Mental Health, Belgrade, Serbia; ^3^Clinic for Gynecology and Obstetrics “Narodni Front”, Belgrade, Serbia

**Keywords:** autism spectrum disorders, Coffin–Siris syndrome, *ARID1B*, developmental disorders, neurodevelopment

## Abstract

**Introduction:**

Autism spectrum disorders (ASDs) are a group of developmental disorders characterized by deficits in social communicative skills and the occurrence of repetitive and/or stereotyped behaviors. Coffin–Siris syndrome (CSS) is classically characterized by aplasia or hypoplasia of the distal phalanx or nail of the fifth and additional digits, developmental or cognitive delay of varying degrees, distinctive facial features, hypotonia, hirsutism/hypertrichosis, and sparse scalp hair. In this study, we present a detailed description of autistic traits in a boy diagnosed with CSS and further discuss their genetic backgrounds.

**Case description:**

An 8-year-old boy with ASD, congenital anomalies, and neurological problems had been diagnosed with Coffin–Siris syndrome after genetic testing. Genetic testing revealed a heterozygous *de novo* pathogenic variant (class 5) c.1638_1647del in the *ARID1B* gene that is causative of Coffin–Siris syndrome but also other intellectual disability (ID)-related disorders, including autism. Tests that preceded the diagnoses, as well as congenital anomalies and developmental issues, were further described in an attempt to better present his phenotype.

**Conclusion:**

Both autism and *ARID1B*-related disorders are on a spectrum. This report points out the importance and necessity of further research regarding the genetic backgrounds of these disorders to understand their complex etiology.

## 1. Introduction

Autism spectrum disorders (ASDs) are a group of developmental disorders with increasing prevalence worldwide. In 2018, one in 44 children aged 8 years was estimated to have ASD (1). The core characteristics of ASD are difficulties in social communication and the occurrence of repetitive and/or stereotyped behaviors. Common ASD-associated impairments include intellectual disability (ID) (currently estimated to occur in ~30% of cases) and attention deficit (occurring in ~30–40% of cases, though estimates outside this range are common), as well as sensory hypo- and/or hypersensitivity, gastrointestinal problems, immune system deficits, anxiety, depression, sleep disturbances (2), motor-skills delays, and a range of other co-occurring conditions (3).

Genetic variation accounts for a major proportion of the liability for ASD. Up to 5–10% of cases can be linked to a known genetic cause via monogenic syndromes (such as Fragile X syndrome, tuberous sclerosis, and Timothy syndrome) (4). Twin studies show that the heritability of ASD is ~50%, given that the concordance of ASD in monozygotic twins ranges from 37 to 95%, depending on the study design and the diagnostic criteria used. The importance of genetics in ASD susceptibility is also reflected by the recent success of microarray and whole-exome sequencing studies, which have established the role of *de novo* copy-number variants (CNVs) and *de novo* protein-disrupting single-nucleotide variants (SNVs) in ASD pathogenesis. Similar contributions have been identified in individuals with ID without ASD (5).

Coffin–Siris syndrome (CSS) is classically characterized by aplasia or hypoplasia of the distal phalanx or nail of the fifth and additional digits, developmental or cognitive delay of varying degrees, distinctive facial features, hypotonia, hirsutism/hypertrichosis, and sparse scalp hair (6).

ARID1B-related disorder (*ARID1B*-RD) constitutes a clinical continuum, from ID with or without non-specific dysmorphic features to classic Coffin–Siris syndrome (7). Heterozygous pathogenic gene variants and cytogenetic abnormalities involving *ARID1B* are considered to be the leading cause of Coffin–Siris syndrome, being found in 68–83% of cases (8, 9). More than 170 unique pathogenic or likely pathogenic variants in *ARID1B* have been reported in ClinVar, most of them *de novo* truncating variants, leading to gene haploinsufficiency (10). What is striking is the considerable clinical variability associated with reduced levels of ARID1B. One review identified the major features associated with ARID1B haploinsufficiency as ID, speech delay, prominent facial features, and hypertrichosis (9). Currently, there is no clear genotype–phenotype correlation, suggesting the involvement of other phenotypic modifiers.

ARID1B is a member of the switch/sucrose non-fermenting (SWI/SNF) chromatin remodeling complex (BAF complex in mammals) and is highly expressed in the brain and embryonic stem cells. It uses an ATP-dependent mechanism to modify the structure of chromatin and change its accessibility to transcription factors. In mammals, these complexes are assembled from subunits encoded by 29 genes, making their composition very diverse (11).

The importance of the BAF complex in human neuronal development and cancer occurrence has emerged through the recent discoveries that mutations in their subunit genes and related genes are implicated in several ID syndromes: CSS, non-syndromic ID (*ARID1B*-MIM: 614556, *ARID1A*-MIM: 603024, *SMARCA4*, BRG1 MIM: 603254, *SMARCB1-*SNF5-MIM: 601607, *SMARCE1*-MIM: 603111, SMARCC2-MIM, *DPF2* MIM: 601671), Nicolaides–Baraitser syndrome (NCBRS, *SMARCA2*-MIM: 600014), sporadic autism, schizophrenia, and amyotrophic lateral sclerosis, as well as in sporadic cancers/cancer-predisposing syndromes (12). Some authors suggest that these disorders caused by mutations in BAF subunit genes represent a clinical continuum with non-syndromic ID and mild CSS on one end of the spectrum and NCBRS on the severe end of the spectrum, suggesting the term SWI/SNF-related intellectual disability disorders (SSRIDDs) (13). Noteworthy, besides mutations in the *ARID1B* gene, Coffin–Siris syndrome can be caused by variants in additional genes, including *ARID1A, ARID1B, ARID2, DPF2, PHF6, SMARCA2, SMARCA4, SMARCB1, SMARCC2, SMARCE1, SOX4*, and *SOX11* (6).

Switches between BAF subunits play an important role in cell fate determination. Recently, it was suggested that two switches between *ARID1A-BAF* and *ARID1B-BAF* in pluripotent stem cells are needed for proper development: first, for differentiation toward the neuroectodermal lineage (*ARID1A* to *ARID1B*) and second for (*ARID1B-*BAF to *ARID1A-*BAF) neuroectodermal sphere differentiation into neural crest cells. Mainly, it was found that the role of the *ARID1B*-BAF subunit is to repress pluripotency enhancers (NANOG/SOX2 networks) during neural crest cell formation, thus enabling exit from pluripotency and lineage commitment. In *ARID1B* haploinsufficient cells, this process is impaired, leading to impaired *ARID1A* subunit activity and dysregulation of neuroectoderm specification, which can explain both neurocognitive and craniofacial characteristic features of CSS (14). Similarly, its haploinsufficiency partially impairs the function of *BRG1*-associated factor complexes, including *BAF155* or *BAF170*. This leads to the dysregulation of C-MYC expression, delaying the cell cycle entry of developmentally arrested cells, such as neural progenitors (15).

This study aims to present a detailed description of ASD manifestations in a boy diagnosed with CSS. Detailed analysis of ASD features in CSS might lead to specific signs that might orient clinicians toward keeping genetic disorders in mind when diagnosing ASD.

## 2. Case description

An 8-year-old boy with developmental disabilities, autistic traits, and neurological problems was diagnosed with Coffin–Siris syndrome after genetic testing in February 2021. He was the first child in his family. Pregnancy was complicated by gestational diabetes, which was treated with a diet. Delivery at term was performed with vacuum extraction. The Apgar score was 9/10, body weight was 3.6 kilograms (P50–P75), and body length was 52 centimeters (P75–P90). He was breastfed for the first 7 months. He had a cow's milk protein allergy, which made him use an adapted milk formula. At 18 months, he started walking without any support, and at 3 years, his walk became more stable. At 4 and 6 months of age, he obtained bladder and bowel control.

He had three surgeries (at 14 months—cryptorchidism, 20 months—strabismus, and 4 years—adenoidectomy). He had febrile convulsions since he was 2 years and 4 months old for which he had taken medication, sodium valproate. EEG measured bitemporal focal changes that were more pronounced on the left side of his slow baseline activity. The development of his language and speech skills was the first cause of parental concern. The delay in this particular domain occurred at the age of 2. This is when they initiated their first referral to the attending physician.

He was examined by an otorhinolaryngologist for problems with speech and for not responding to commands when he was 2 years and 1 month old. Brainstem-evoked response audiometry (BERA) testing had been performed, and there were no pathological findings. He was assessed by a child psychiatrist on suspicion of autism 1 month later and diagnosed with delayed psychomotor development. Both mother and father denied any family history of significant hereditary disorders. He was referred to our Institution for detailed diagnostic exploration when he was 5 years old.

Physical examination showed that stature and head circumference were age-appropriate. Other findings included facial dysmorphia and macrostomia with small, widely spaced teeth, long eyelashes, downslanted palpebral fissures, lower placed hairline with thick and coarse hair, pronounced and descended lower lip, single palmar crease on one hand, and fifth digit distal phalangeal hypoplasia on his feet, as well as a protosystolic heart murmur. According to his parents, diagnostic imaging did not reveal any congenital structural anomalies of the brain or abdomen.

On psychiatric examination, it was noted that the child was well-nourished, active, and curious. A greeting gesture was noted. Eye contact was brief. He immediately approached the toy boxes and spent some time with each of them. Contact with the boy was successfully achieved through playing with toys. He accepted organized play (driving a teddy bear with a toy car). He did not develop any functional speech. Stereotypical movements were not observed. He was clumsy, poorly coordinated, and his sense of spatial orientation was insufficient. It was stated that he participated in organized play, but only briefly. He enjoyed the company of children and tried to interact with them. He particularly enjoyed activities that include motor skills: climbing, jumping, and running. He did not eat with a fork and a knife and did not drink from a glass on his own. He could take off his shoes without any assistance.

A speech therapist examination showed that contact through toys and other objects was achieved with difficulties. Contact through voice and words was not made. He was not turning his head toward the examiner when he was called by his name. Motor skills in total were underdeveloped for his age. Movement of the upper and lower extremities was uncoordinated. His body was often bent forward, which could be attributed to his poor eyesight. Play routines were simple, with very little imagination. He solved the wooden puzzle with animals, letters, and terms without deepening his interaction with the examiner. He played alone and did not show any interest in playing with others. He did not speak. He used two words in the form of syllables with the goal of expressing his needs. Contrary to the psychiatrist's initial assessment, the greeting gesture was confirmed to be absent. He was constantly moving, walking around with toys in his hands, and had difficulty focusing on activities outside of his scope of interest.

His score for general adaptive behavior was 58 on the Vineland II Adaptive Behavior scale. A delay in psychomotor development with significant difficulties in communication (underdeveloped speech and difficulties in comprehension), socialization, and motor skills was noted. The childhood autism rating scale (CARS) was used for psychometric assessment. A score of 29 (borderline value) was noted.

The comprehensive and multidisciplinary team assessment led to the conclusion that his developmental difficulties were significant. There were autistic traits, developmental delay, and neurological problems (seizures). He was formally diagnosed as having ASD by a child psychiatrist with supporting evidence from the Autism Diagnostic Interview-Revised (ADI-R) and the Autism Diagnostic Observation Schedule 2 (ADOS-2). The ADI-R scores were as follows: Total A−21; Total non-verbal B−11; Total C−8; and Total D−5. His total score on Module 1 of ADOS-2 was 22 (Table 1), which was beyond the cutoff criteria for autism. The detailed family history revealed that there were no developmental, neurological, or psychiatric disorders in the family.

**Table 1:** ADOS-2 scoresheet.

**Social affect (SA)**	
**Communication**	
Frequency of spontaneous vocalization directed to others	2
Pointing	1
Gestures	2
**Reciprocal social interaction**	
Unusual eye contact	2
Facial expressions directed at others	1
Integration of gaze and other behaviors during social overtures	2
Shared enjoyment in interaction	1
Showing	2
Spontaneous initiation of joint attention	2
Response to joint attention	1
Quality of social overtures	2
**Restricted and repetitive behavior (RRB)**	
**Restricted and repetitive behaviors**	
Stereotyped/idiosyncratic use of words or phrases	0
Unusual sensory interest in play material/person	2
Hand and finger and other complex mannerisms	0
Unusually repetitive interests or stereotyped behaviors	2
Overall total (SA + RRB)	22

Since the boy presented with ASD, congenital anomalies, and specific features, genetic testing was performed. Karyotype, Fragile X syndrome testing, chromosomal microarray analysis, and urine metabolite screening did not detect any genetic abnormalities. However, the Trio whole-exome sequencing showed a heterozygous *de novo* variant in the *ARID1B* gene (NM_001371656.1):c.1638_1647del that was classified as pathogenic (class 5 according to ACMG/AAP criteria). This is a frameshift variant leading to non-sense-mediated decay of mRNA. Heterozygous loss of function variants in this gene are causative of Coffin–Siris syndrome.

The boy had attended several different stimulation treatments during the past few years, including logopedic, psychomotor re-education, and sports activities, which have resulted in improvements in several domains. The comprehension of speech and articulation of syllables have improved. He got better at verbalizing his needs. He has a personal assistant and is attending second-grade school for children with special needs, but is attending math classes under a regular program. Aside from verbalization, his other biggest difficulty is graphomotorics. Verbalization still includes only several words. He is of adequate behavior at home. He still needs support and stimulation with his everyday activities, including dressing up, tying his shoes, eating, and maintaining his personal hygiene.

## 3. Discussion

In this study, we presented a detailed description of a patient who had typical clinical manifestations of CSS (dysmorphic features, strabismus, cryptorchism, hypertrichosis, and seizures) (7) and the core clinical features of ASD. A large study by van der Slujis (10) found that among 79 patients with *ARID1B* mutations and features of CSS, 66.7% had ASD. A study that examined the abilities of 12 children with CSS found that symptoms of pervasive developmental disorders (PDDs) were found in almost half of the CSS patients (16). We compared the features and mutations of these cases with ours, as shown in Table 2.

**Table 2:** Comparison of cases with ASD and CSS and their mutations.

**Cases**	**Characteristics**	**Mutations in CSS**
Our case	Not responding when called by name. Brief participation in organized play, play routines were simple with very little imagination. He played alone and did not show any interest in playing with others. He did not speak. He used two words in the form of syllables with the goal of expressing his needs. A greeting gesture was absent. Difficulty focusing on activities outside of his scope of interest. Poor sense of coordination and spatial orientation. • In summary, our case was presented with underdeveloped speech, difficulties in comprehension, socialization, and motor skills.	*ARID1B*
Seda Gulcu et al. (17)	Very little social interaction, restricted facial expression. Limited eye contact, failure to look when called, the absence of focus of interest, unwillingness to point to any desired object, lack interest in his peers, and “flapping” motions when excited.	*ARID1B*
Hersh et al. (18)	Delayed speech development. Only vocalizations heard during the evaluation were lip-trilling sounds. Only interaction with the examiner was pushing his hand toward an object she wished to obtain. Non-existent eye contact, self-occupying behavior. Functional play was not observed.	Genetic tests were not performed
Krause and Rose (19)	The patient was described as having difficulties with expressive speech and speech impairment (despite this, verbal abilities were a relative strength in his cognitive profile), diverted eye contact, restricted interests and stereotypical behaviors, inappropriate social gestures, idiosyncratic and pedantic speech, and restricted affect.	Genetic tests were not performed

When it comes to clinical characteristics of ASD symptoms in our case, the most severe symptoms were seen in the disturbance of reciprocal social interaction (eye contact, imaginative play with peers, and lack of need to share pleasure with others) and qualitative communication disturbances (pointing to show interest and nodding). For the restrictive, repetitive, and stereotyped behaviors, the boy showed a lower presence and severity of symptoms, and mostly for stereotyped and repetitive hand and finger mannerisms. It would be potentially beneficial to explore whether there might be a specific clinical phenotype in children with ASD as part of CSS.

ARID1B (MIM135900), besides being a disease-causing gene in CSS, is a prevalent underlying genetic cause of ID (0.5–1%) and ASD (15). Analysis of rare coding variation in 3,871 ASD cases and 9,937 ancestry-matched or paternal controls identified *ARID1B* (20) as a strong candidate to be an ASD risk gene based on a combination of *de novo* mutational evidence and the absence or very low frequency of mutations in controls (21). The deletion of 10 bp found in our patient is located in exon 2 of the ARID1B gene; however, the location of the pathogenic variant does not appear to correlate with the severity of the phenotype (22). The pathogenic gene variant found in our patient was published by Tsurusaki et al. (23), but no detailed description of the patient's characteristics was given.

Recently, models of *ARID1B* heterozygous mutant mice reportedly expressed social behavior impairment, altered vocalization, motor abnormalities, and neuroanatomical anomalies, including agenesis of the corpus callosum (24). A recent morphometric analysis reveals that children with ASD usually have a smaller corpus callosum compared to neurotypical children of the same ethnicity (25).

Many neurodevelopmental disorders exhibit improper inhibitory interneuron development, resulting in excitatory/inhibitory imbalance, including ASD (26, 27). One study showed that *ARID1B* haploinsufficient mice exhibit a reduction and abnormal distribution of interneurons as well as abnormal inhibitory synaptic activity in the cerebral cortex (28). It was also found that *ARID1B* haploinsufficiency in parvalbumin- or somatostatin-expressing interneurons lead to distinct ASD-like and ID-like behaviors (29).

Finally, *ARID1B* belongs to a group of genes that encode key regulators of chromatin remodeling, altering translation within neurons and at synapses. A better understanding of pathways such as this one might lead to a more specific, core autistic symptom-oriented treatment (30). Furthermore, due to the identification of the specific syndrome underlying the symptoms the boy presented with, we were able to advise the parents on future regular somatic checkups, such as the evaluation of growth, endocrinological, and vision and hearing evaluation (22).

The limitation of this study is that it is focused on a single clinical case and, therefore, provides little basis for generalizing results to a wider population. However, it highlights the importance of genetic testing for ASD patients in order to determine the possible underlying cause of this complex condition, which can lead to better understanding and acceptance.

## 4. Conclusion

*ARID1B*-related disorder, such as autism, is on a spectrum (22). Similarities between the two can be explained by the fact that mutations in both disorders are related to genes associated with neuronal communication, synapse development, and chromatin remodeling/transcription regulation. This points out the importance and necessity of further research regarding the genetic background of these disorders in order to understand their complex etiology. Furthermore, this study could serve as a reminder for clinicians to always be aware of genetic syndromes hiding behind the common ASD diagnosis. Genetic consultation can help identify genetic syndromes and other frequent comorbidities in ASD. Knowing the underlying cause of ASD can be very beneficial for parents and families of children with ASD.

## Data availability statement

The raw data supporting the conclusions of this article will be made available by the authors, without undue reservation.

## Ethics statement

Ethical approval was not required for the study involving human samples in accordance with the local legislation and institutional requirements. Written informed consent for participation in this study was provided by the participants' legal guardians/next of kin. Written informed consent was obtained from the individual(s) for the publication of any potentially identifiable images or data included in this article.

## Author contributions

LM participated in the literature review, writing the manuscript, and data collection. RG participated in writing the manuscript, literature review and preparation, revising the manuscript, and testing the patient. VMM participated in revising the manuscript and data collection. IJ participated in revising the manuscript and contributed genetic expertise. NL participated in revising the final version of the manuscript and literature research. MPM recruited the patient, obtained informed consent, revised the manuscript, and supervised the research. All authors contributed to the article and approved the submitted version.
